# The patterns, trends and major risk factors of suicide among Indian adolescents—A scoping review protocol

**DOI:** 10.1371/journal.pone.0277422

**Published:** 2022-11-17

**Authors:** Rachel Elizabeth Senapati, Jayashree Parida, Jagatdarshi Badamali, Susangita Jena, Pranita Patsani, Arpita Panda, Swati Sukalyani Behera, Abinash Pradhan, Prashant Kumar Singh, Bijaya Kumar Mishra, Prasanna Kumar Patra, Sanghamitra Pati, Harpreet Kaur, Subhendu Kumar Acharya

**Affiliations:** 1 ICMR-Regional Medical Research Centre, Bhubaneswar, Odisha, India; 2 ICMR- National Institute of Cancer Prevention and Research, New Delhi, India; 3 Department of Anthropology, Utkal University, Bhubaneswar, Odisha, India; 4 Division of ICMR, Division of Epidemiology and Communicable Diseases (ECD-Tribal Health), New Delhi, India; Sapienza University of Rome: Universita degli Studi di Roma La Sapienza, ITALY

## Abstract

**Introduction:**

Suicide is one of the serious health problems among Indian adolescents. Adolescence helps with the transition of an individual into an adult, so it is important to understand the suicidal behavior of adolescents. Several studies have been carried out in different states in India on the suicidal behavior of adolescents, but there is no review that studied the national patterns, trends, and major risk factors. Therefore, this review aims to study the patterns, trends, and major risk factors of suicidal behavior among Indian adolescents.

**Methods:**

The study will be conducted as per the Arksey and O’Malley scoping review framework and the Joanna Briggs institute Reviewers’ manual. The Population, Concept and Context strategy (PCC) will ensure the review questions, eligibility criteria and search strategy. The Systematic Review and Meta-analysis: Extension for Scoping Review (PRISMA-ScR) will be used for the findings of the study of Scoping Review. The literature search will be done using electronic databases: PubMed, Google Scholar, SCOPUS, EMBASE, Psycinfo, Web of Science, Google, and Cochrane library by specific keywords such as “patterns”; “suicide”; “trends”; “risk factors”; “depression”; “anxiety”; “mental health”; “adolescent”; “teenager”; and “youth”;” India” etc. Additional studies will be considered using cross-references.

**Ethics and dissemination:**

This study does not involve the collection of primary data; there is no requirement of any ethical approval.

**Strengths and limitations of the study:**

- The proposed scoping review is the first study on patterns, trends, and major risk factors of adolescent suicide in India as per the information available on the online platforms.
- This proposed scoping review will bring together all the previously available data into one place for better study.
- This evidence-based study will be highly helpful for healthcare professionals and policymakers. This study will be limited to English language and electronically available evidence.

## Introduction

Suicide is one of the major mental health concerns that is found in every society across the world [1]. Suicide is the intentional or deliberate act of killing own self [2]. Globally, suicide is the 15^th^ leading cause of death for all ages [3, 4]. Despite the commendable progress of human society in the 21^st^ century, one person dies due to suicide in every 40 seconds. Across the world nearly around 8 hundred thousand suicide cases are registered every year and out of them, 77% suicides occur in low -and -middle income countries [5]. It has been observed that with a suicide rate of 10.5/100,00 (for the world, it is 11.6/100,00), India reports a concerning suicide prevalence [6]. According to Indian National Crime Records Bureau (NCRB), the numbers of suicides in India in the years 2019 and 2020 reportedly increased by 10% and 8.7% compared to the previous years respectively [7]. Suicide among Indian adolescents constitutes a significant proportion of the total number of suicides every year in the country. In India population of adolescents is the largest as it constitutes one-fifth population in India [8].

Adolescence in India is defined as the age group ranging between 10–19 years [9]. Studies show that adolescents in India have consistently been highly vulnerable to suicide [10] due to the early onset of various complexities in their lives leading to ambiguity and lack of experience to handle pressure pushing them into the trap of quick escape and committing suicide [11]. In this phase, many physical and psychological developments including hormonal changes leading to adulthood occur [9]. Findings suggest suicide is the 4^th^ leading cause of death among the late adolescent age group [12]. According to NCRB 2020 report, 11396 adolescents (below 19 years of age) died due to suicide in the previous year [13]. More broadly, mental health conditions are a major risk factor in adolescent age group. According to UNICEF, an estimated 166 million adolescents (89 million boys and 77 million girls) had mental health conditions, which means one in seven adolescents experience mental health disorders [14]. So, in this context, it is crucial to ensure that adolescents are fully supported and cared for in all aspects of life, including their mental health and well-being so that they can smoothly lay a foundation for their healthy and productive adulthood.

To improve and break the cycle of adolescent suicide, there is a need of evidence-based study, and this proposed scoping review will fulfil this aspect by providing the knowledge about the pattern of suicide by focusing on the methods, time and location of suicide attempt which will help to devise suicide preventive strategies [15]. Our next objective is to analyze how suicide study is changing over time and analyze its trend which will help decision-makers to understand whether the current strategies are working or not [16]. Suicide risk factors are the things that has the potential to increase the chances of suicide hence understanding of the risk factors will help us to counteract the fact that suicide is a random act or is a result of just stress or depression [17]. Several studies evaluated adolescent anxiety [18], depression [19], and suicidal behavior [20] but no review, as per our knowledge, is available having explained the patterns, trends, and risk factors associated with suicidal behavior in Indian adolescents. Therefore, the planned scoping review aims to provide the patterns, trends, and major risk factors associated with suicidal behavior in Indian adolescents which can help further research and policy-making.

## Objectives

The objectives of the planned scoping review are as follows:

- To map the patterns and trends of suicide among the adolescents of India.
- To understand the major risk factors associated with suicide among the adolescents of India.

## Methods and analysis

In the present review, we aim to synthesis the evidence around selected objectives defining the patterns, trends and the major factors associated with suicide among the adolescents of India. Prior to defining the objectives and design of the study, we first checked for the availability and accessibility of any scoping review on suicidal status among the adolescent population of India in an online database and protocol registration website. We checked it on Figshare, Open Science Framework; consequently, we found that there is no previously published scoping review on suicide among adolescents of India, followed by which the present study was designed.

This study will be conducted according to the Arksey and O’Malley scoping review framework (Arksey and O’Malley, 2005) [21] and will also follow the recommendations on writing a scoping review given by the Joanna Briggs Institute Reviewers’ Manual [22] and Levac et.al [23]. The Arksey and O’Malley scoping review framework includes five stages to conduct a scoping review.

1. Identifying the research questions,
2. Identifying relevant studies,
3. Study selection,
4. Charting the data,
5. Collating, summarizing, and reporting results.

The research questions, search strategy and eligibility criteria will be conducted based on the Population, Concept and Context (PCC) strategy [22].

### Research questions

Following research questions are framed according to the Population, Concept, and Context (PCC) strategy:

- What are the patterns and trends of suicide among the adolescent population in India?
- What are the relevant risk factors affecting adolescent suicide in India?

### Identifying relevant studies

At this stage, the relevant studies will be identified and collected through extensive literature search. The reviewers will identify the relevant key words and undertake the search of research papers. Further, the inclusion and exclusion criteria for selecting studies will also be defined according to the PCC strategy. The details procedure will be carried out in a step-by-step manner which has been discussed in the following sections:

#### Search strategy

The present review will collect relevant literature which will be identified from different online databases such as PubMed, EMBASE, PsycInfo Google Scholar, Web of Science, and Google. This scoping review literature search will be performed by using the various keywords such as, “patterns”; “suicide”; “trends”; “risk factors”; “depression”; “anxiety”. “Mental health”; “suicidal tendency”; “adolescents”; “teenager”; “youth”; and “India” etc. The strategy used for searching PubMed electronic database is given in S1 File. All the grey literature, Govt. reports, short communication, and editorial letters will be considered for the review. The reference list of the articles will also be searched to find relevant articles.

#### Eligibility criteria

*Inclusion criteria*.

- Population: This review will consider all studies focusing on suicide among the adolescent group ranging between 10–19 years of age which is defined by the World Health Organization. There is no gender specificity included in the study.
- Concept: For the first research question, various patterns regarding the methods, location and time of suicide will be explored and the trend of the patterns observed will be evaluated.

And for the second question, the precipitating influencing factors that act as the risk factors for suicide will be studied.

This review will consider quantitative study designs reporting the patterns, trends, and major risk factors of suicide among Indian adolescents. Any qualitative study design that investigates the experiences before or after attempting suicide will also be considered.

- Context: This review will consider studies in the Indian context only.

*Exclusion criteria*.

- Studies were undertaken prior to the year 2000 or considering populations other than India.
- Studies conducted in languages other than English will be excluded.

### Study procedure and selection of the studies

To ensure the comprehensiveness of the study, screening and selection procedure of the articles will be shadowed from the review. Titles and abstracts of the original articles will be assessed, and duplicate articles will be removed from the study by the investigators followed by cross check by the reviewers. The already defined inclusion and exclusion criteria will be followed to select the relevant literature. The records identified by the reviewers will be included in the full-text screen; the same eligibility criteria will be followed to screen the full-text article.

In this context, if any disagreement arises among the reviewers that will be resolved by mutual discussion or by referring to the third reviewer. All the stages for selecting the relevant studies will be presented in the flow diagram as prescribed in Preferred Reporting Items for Systemic Review and Meta-analysis Scoping Reviews (PRISMA-ScR) [24].

### Charting the data

The reviewers will extract the relevant data from the selected literature. A predefined data extraction form will be developed using Microsoft-Excel spread sheet. A summary table will be presented consisting of the key information of the selected studies. This information will include title of paper, year, journal, author, area of study, residency, region, type of study, sample size, age, group, gender, methods, mode of suicide, and risk factors.

Before finalization, all the feedback from the investigators will be considered to update the data extractions from the included studies.

### Collating, summarizing, and reporting the results

As the aim of the proposed scoping review is to collect the existing evidence on the patterns, trends, and major risk factors of adolescent suicide, summarize the results as reported in the included studies and identify gaps for further research, all the processed evidence as discussed above will be proceeded to summarization, analysis and preparing the final report.

The findings will be analyzed with thematic content analysis method. The data were extracted manually to the relevant things. The themes will be collated, summarized, and reported around the following outcomes: patterns, trends, and the major risk factors of suicide. Other emerging themes will be reported.

### Ethics and dissemination

Scoping review does not require ethical approval; it involves a systematic combination and presentation of available resources. This protocol will provide an exceptional overview of the literature available on the adolescent suicide in India. The proposed scoping review will systematically map the evidence to identify research priorities and to uncover the research gaps around adolescent suicide issues in India. The potential gaps will provide essential inputs to policymakers and various healthcare agencies to outline new research questions and interventions to improve adolescent health in India.

## Discussion

Adolescents are very vulnerable to suicide in India which acts as a major public health challenge for the country. India has the largest adolescent population in the world having 253 million adolescents and if this large number of adolescents become safe, healthy, educated, and equipped with information and life skills then India will be benefited for development in the social, political, and economic context [25]. The incidence of adolescent suicidal behavior in India shows a rising trend day by day [26], hence the study of suicide in the Indian context holds a vital place.

The previous reviews conducted on suicidal behavior [27, 28] at national and local levels have focused more on the adult and older age groups hence they lack relevant data focusing majorly on adolescents. The accumulation and summarization of the information on the suicidal behavior of adolescents can be beneficial for a better understanding of the patterns and trends of suicidal behavior of Indian adolescents and it can also explain how the major risk factors influence the patterns and trends of suicide. This review will provide helpful information for further research as well as for the healthcare practitioners. This summarized finding at the national level will help to make better mental health awareness, to build confidence, to seek help and to provide proper medical services so that the suicide rate could be prevented to a greater extent and lives at risk of suicide would be saved.

## Supporting information

S1 File Search strategy for PubMed electronic database. (DOCX)

S1 Checklist (DOCX)
