# Supplementary material for: The patterns, trends and major risk factors of suicide among Indian adolescents—A scoping review protocol
Source: PLoS One. 2022 Nov 17;17(11):e0277422. doi: 10.1371/journal.pone.0277422 (PMC9671363; doi:10.1371/journal.pone.0277422)
Supplement: S1 File — (DOCX) [file pone.0277422.s001.docx]

**Draft of search strategy to be used using PubMed electronic database**

| **Components** | **Search items** | **Results** |
| --- | --- | --- |
| #1 |  |  |
| #2 | **adolescents:** "adolescences"[All Fields] OR "adolescency"[All Fields] OR "adolescent"[MeSH Terms] OR "adolescent"[All Fields] OR "adolescence"[All Fields] OR "adolescents"[All Fields] OR "adolescent's"[All Fields] | **2,260, 921** |
| #3 | **stunting:** "growth disorders"[MeSH Terms] OR ("growth"[All Fields] AND "disorders"[All Fields]) OR "growth disorders"[All Fields] OR "stunting"[All Fields] OR "stunted"[All Fields] | 103,011 |
| #4 | **underweight:** "thinness"[MeSH Terms] OR "thinness"[All Fields] OR "underweight"[All Fields] OR "underweights"[All Fields] | 18,782 |
| #5 | **Indian:** "Indian"[All Fields] OR "Indian's"[All Fields] OR "Indians"[All Fields] | 31,652 |
| #6 | **Teenager:** "adolescent"[MeSH Terms] OR "adolescent"[All Fields] OR "teenage"[All Fields] OR "teenager"[All Fields] OR "teenagers"[All Fields] OR "teenaged"[All Fields] OR "teenager's"[All Fields] OR "teenages"[All Fields] | 2,220,471 |
| #7 | **Young adults:** "young adult"[MeSH Terms] OR ("young"[All Fields] AND "adult"[All Fields]) OR "young adult"[All Fields] OR ("young"[All Fields] AND "adults"[All Fields]) OR "young adults"[All Fields] | 1,188,667 |
| #8 | **#2 AND #3 AND #4 AND #5**  "growth disorders"[MeSH Terms] OR ("growth"[All Fields] AND "disorders"[All Fields]) OR "growth disorders"[All Fields] OR "stunting"[All Fields] OR "stunted"[All Fields]) AND ("thinness"[MeSH Terms] OR "thinness"[All Fields] OR "underweight"[All Fields] OR "underweights"[All Fields]) AND ("indian"[All Fields] OR "indian s"[All Fields] OR "indians"[All Fields]) AND ("adolescences"[All Fields] OR "adolescency"[All Fields] OR "adolescent"[MeSH Terms] OR "adolescent"[All Fields] OR "adolescence"[All Fields] OR "adolescents"[All Fields] OR "adolescent s"[All Fields]) | 66 |
| #9 | **#1 AND #2 AND #5**  ("malnutrition"[MeSH Terms] OR "malnutrition"[All Fields] OR "undernutrition"[All Fields] OR "undernutritional"[All Fields]) AND ("indian"[All Fields] OR "indian s"[All Fields] OR "indians"[All Fields]) AND ("adolescences"[All Fields] OR "adolescency"[All Fields] OR "adolescent"[MeSH Terms] OR "adolescent"[All Fields] OR "adolescence"[All Fields] OR "adolescents"[All Fields] OR "adolescent s"[All Fields]) | 634 |
| #10 | **#1 AND #6 AND #2 AND #5**  ("malnutrition"[MeSH Terms] OR "malnutrition"[All Fields] OR "undernutrition"[All Fields] OR "undernutritional"[All Fields]) AND ("adolescent"[MeSH Terms] OR "adolescent"[All Fields] OR "teenage"[All Fields] OR "teenager"[All Fields] OR "teenagers"[All Fields] OR "teenaged"[All Fields] OR "teenager s"[All Fields] OR "teenages"[All Fields]) AND ("indian"[All Fields] OR "indian s"[All Fields] OR "indians"[All Fields]) AND ("adolescences"[All Fields] OR "adolescency"[All Fields] OR "adolescent"[MeSH Terms] OR "adolescent"[All Fields] OR "adolescence"[All Fields] OR "adolescents"[All Fields] OR "adolescent s"[All Fields]) | 622 |
| #11 | **#1 AND #7 AND #2 AND #5**  ("malnutrition"[MeSH Terms] OR "malnutrition"[All Fields] OR "undernutrition"[All Fields] OR "undernutritional"[All Fields]) AND ("young adult"[MeSH Terms] OR ("young"[All Fields] AND "adult"[All Fields]) OR "young adult"[All Fields] OR ("young"[All Fields] AND "adults"[All Fields]) OR "young adults"[All Fields]) AND ("indian"[All Fields] OR "indian s"[All Fields] OR "indians"[All Fields]) AND ("adolescences"[All Fields] OR "adolescency"[All Fields] OR "adolescent"[MeSH Terms] OR "adolescent"[All Fields] OR "adolescence"[All Fields] OR "adolescents"[All Fields] OR "adolescent s"[All Fields]) | 144 |
